# Excellent long-term outcomes of endovascular treatment in budd–chiari syndrome with hepatic veins involvement: A STROBE-compliant article

**DOI:** 10.1097/MD.0000000000012944

**Published:** 2018-10-26

**Authors:** Yonghua Bi, Hongmei Chen, Pengxu Ding, Pengli Zhou, Xinwei Han, Jianzhuang Ren

**Affiliations:** a Department of Interventional Radiology, The First Affiliated Hospital of Zhengzhou University; b Department of Ultrasound, Zhengzhou Central Hospital Affiliated to Zhengzhou University, Zhengzhou, China.

**Keywords:** balloon angioplasty, Budd–Chiari syndrome, hepatic veins, inferior vena cava, transjugular intrahepatic portosystemic shunt

## Abstract

This study aimed to evaluate the long-term efficacy and safety of percutaneous transhepatic balloon angioplasty (PTBA) and transjugular intrahepatic portosystemic stent-shunt (TIPSS) in the treatment of Budd–Chiari syndrome (BCS) with hepatic veins involvement. Between June 2008 and August 2016, a total of 60 BCS patients with hepatic vein involvement in our department were enrolled in this study. Thirty-three cases underwent hepatic vein balloon angioplasty in PTBA Group and 27 cases underwent TIPSS. Data were retrospectively collected, and follow-up observations were performed. TIPSS Group showed significantly higher thrombotic/segmental obstruction and peripheral stenosis/obstruction compared with PTBA Group. The success rates were 93.9% and 100.0% in PTBA Group and TIPSS Group, respectively. The mean portal vein pressure decreased significantly after stenting. Except for 1 patient died from repeated hemorrhage, other sever complications had not been observed in both group. Twenty-six patients and 21 patients were clinically cured in PTBA Group and TIPSS Group, respectively. The primary patency rates were 89.7%, 79.3%, and 79.3% for short-term, mid-term and longterm in PTBA Group, which were significantly higher than TIPSS Group for long-term follow up. The second patency rates were 100.0%, 96.6% and 96.6% for short-term, mid-term and long-term in PTBA Group, which were similar to TIPSS Group (*P* = 1.0000). In conclusion, PTBA and TIPSS are safe and effective in the treatment of BCS with hepatic veins involvement, with an excellent long-term patency rate of hepatic vein and TIPSS shunt. TIPSS can be used to treat patients with all 3 hepatic veins lesion and failure PTBA.

## 1 Introduction

Budd–Chiari syndrome (BCS) is a rare disease in European and American countries.^[1]^ The hepatic vein stenosis or occlusion due to thrombosis is the main cause,^[2,3]^ shunt operation is the common treatment and liver transplantation is further needed.^[4,5]^ To the contrary, the majority of BCS in China are mixed type of hepatic veins and inferior vena cava (IVC) involvement, where membranous obstruction predominates.^[3]^ Percutaneous transhepatic balloon angioplasty (PTBA) is the main treatment in a population that differs from the US population. BCS with hepatic veins involvement is manifested by severe liver damage in acute cases and liver cirrhosis, portal hypertension and esophageal variceal bleeding or refractory ascites in chronic cases,^[6]^ which is the focus and difficulty of interventional therapy. Nowadays, PTBA of hepatic veins and transjugular intrahepatic portosystemic stent-shunt (TIPSS)^[1,7–15]^ have become the main treatments for BCS with hepatic vein involvement. Few studies compared the long-term outcomes of PTBA and TIPSS.^[8]^ The aim of this study was to evaluate the long-term efficacy and safety of PTBA and TIPSS in the treatment of BCS with hepatic veins involvement.

## 2 Materials and methods

This retrospective study was approved by the ethics committee of Zhengzhou University; all procedures were performed in accordance with the guidelines and regulations for clinical study. All informed consents were obtained from individual participants enrolled in this study.

### 2.1 Clinic data

Between June 2008 and August 2016, a total of 60 BCS patients with hepatic involvement in our department were enrolled in this study. Contrast-enhanced CTA or magnetic resonance angiography, and color Doppler ultrasonography were required in all patients after admission, followed by hepatic vein and IVC angiography during interventional procedure. The decision making process to route patients to PTBA or TIPSS is depending on venous disease characteristics. TIPSS was chosen for patients with all 3 hepatic veins lesion or failure PTBA. Patients with severe liver function failure, terminal hepatic carcinoma, or missing clinical data were excluded from this study. Patients were excluded if they already had a previous TIPSS revision or a parallel shunt due to failed original shunt revision.

### 2.2 PTBA

**Figure 1:**
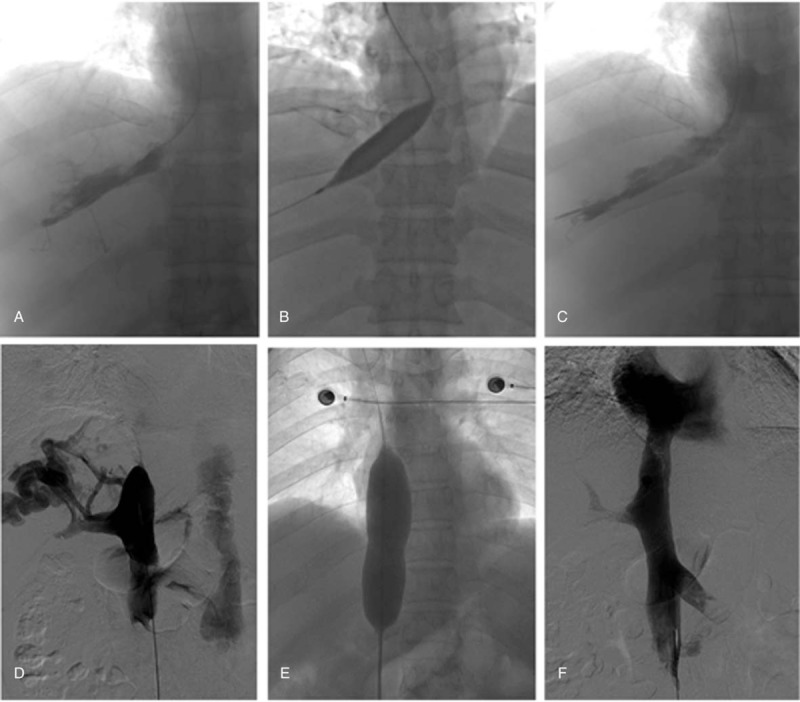
Balloon angioplasty was performed for hepatic vein and IVC. (a) The segmental obstruction in hepatic vein was shown via angiography. (b) PTBA performed by using a balloon of 14 mm in diameter. (c) Patency of hepatic vein was confirmed by second angiography. (d) Proximal segment occlusion of IVC was shown via angiography. (e) A balloon of 26 mm in diameter was used for dilation. (f) Second angiography showed the patency of IVC. IVC = inferior vena cava, PTBA = percutaneous transhepatic balloon angioplasty.

All patients underwent right femoral vein and/or right internal jugular vein puncture and catheterization after local anesthesia. The hunter head catheter was catheterized to the lesion (accessory) hepatic vein for PTBA. If catheterization failed, percutaneous transhepatic puncture was used. The 21G platinum needle wire was introduced into the vein after puncture via a 21-G Chiba needle. The occlusive/stenostic segments of (accessory) hepatic veins were dilated by using a balloon of 6 to 18 mm diameter and 40 to 60 mm length (Fig. 1).

### 2.3 TIPSS

**Figure 2:**
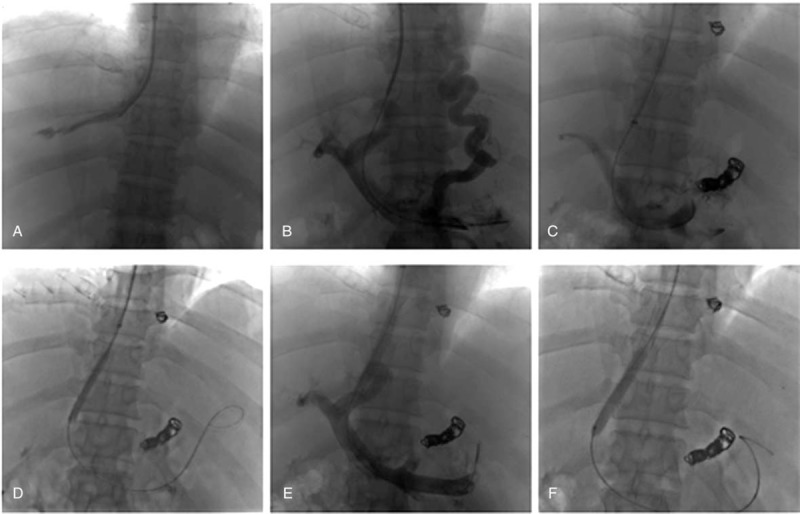
TIPSS procedure. (a) Hepatic vein was catheterized. (b) The main portal vein was punctured by the Rupss-100 puncture device. (c) The esophageal and gastric varices should be embolized. (d) A balloon catheter of 6 mm diameter and 40 mm length was used to dilate the shunt. (e) A covered metal stents of 8 mm diameter and 60 mm length were implanted, and second angiography performed to show the patency of shunt. (f) Post-dilation performed with a balloon catheter of 10 mm diameter.

The right internal jugular vein was punctured and the Rupss-100 puncture device was introduced through the hard guide wire. The main portal vein was punctured after adjusting the angle of intrahepatic puncture according to the anteroposterior images. A balloon catheter of 6 to 10 mm diameter and 40 to 80 mm length was used to dilate the shunt, then bare stent E-Luminexx stent (Bard Peripheral Vascular, Tempe, Arizona) and/or covered Fluency stents (Bard Incorporated, Karlsruhe, Germany) of 8–10 mm diameter and 40 to 80 mm length were implanted (Fig. 2).

### 2.4 Postoperative medicine and follow-up

**Figure 3:**
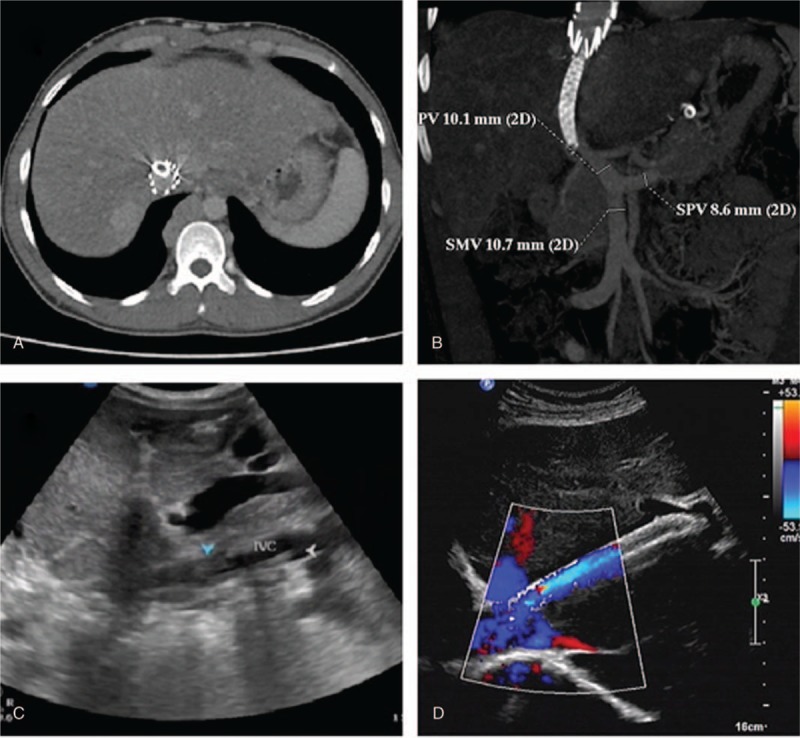
CTA and color Doppler ultrasonograpy test after procedure. The stent shunt and stent in IVC were shown by CTA in coronal (a) and sagittal position (b). The color Doppler ultrasonograpy was used to exam the patency of IVC (c) and stent shunt (d) during follow up. CTA = computerized tomography angiography, IVC = inferior vena cava.

A 5100U low molecular weight heparin was subcutaneous injected every 12 hours for 4 ∼ 5 days. All patients underwent anticoagulation therapy by oral take of warfarin for at least 1 year, with international normalized ratio between 2 and 3. Hepatic vein and shunt patency was evaluated by Doppler ultrasound about 1 week after procedure to avoid acute complications. Re-evaluation was performed by Doppler ultrasound with or without computerized tomography angiography (CTA) every 6 months thereafter (Fig. 3). Color Doppler or CTA examination revealed stenosis of the TIPSS shunt. Accelerated blood flow is defined as recurrence of restenosis. The curative effect of short-term (within 1 year), mid-term (1–3 years) and long-term (more than 3 years) were recorded. Clinical efficacy was defined as below. Cured: patency in hepatic vein/shunt without clinical symptoms; Improved: hepatic vein/shunt diameter is less than 5 mm without obvious clinical symptoms. Invalid: hepatic vein/shunt obstruction and clinical symptoms worsen again.

### 2.5 Statistical analysis

Data were expressed as mean ± standard deviation, and analyzed by student *t* test and analysis of variance (ANOVA). Qualitative data were expressed in percentage, and analyzed by Fisher exact test. Patency rates were analyzed and compared using the Kaplan–Meier method with Log-rank (Mantel-Cox) Test (GraphPad Software, Inc.). Statistical significance was considered when *P* < .05.

## 3 Results

### 3.1 Patient characteristics

A total of 60 BCS patients were included in the study. Thirty-three cases underwent hepatic vein balloon angioplasty in PTBA Group (23 men; median age 38 years), and 27 cases underwent TIPSS (15 men; median age 47 years), including 1 failure PTBA and 1 recurrent patient after PTBA for hepatic veins, who treated in other centers. Patient characteristics are almost similar between 2 groups, except that more patients showed a symptom duration between 0.5 and 3 years in TIPSS Group (*P* = .0342). About half of patients showed a symptom less than 0.5 year in PTBA Group. Abdominal distension and pain was the most common symptom in both group, and gastrointestinal bleeding was more common in TIPSS Group, although there was not significant (Table 1).

**Table 1:**
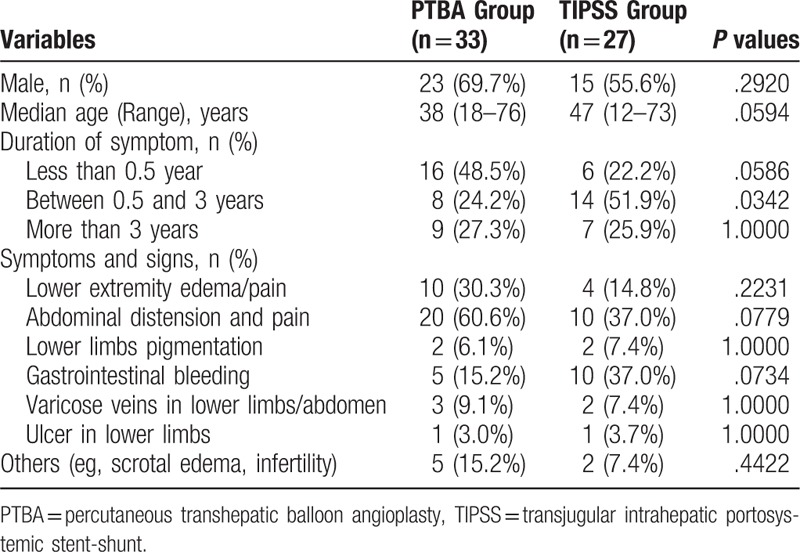
Patient demographics and clinical characteristics.

### 3.2 The type and classification of BCS

As shown in Table 2, 15 cases (45.5%) had combined hepatic vein and IVC involvement, and 18 patients had isolated hepatic vein involvement in PTBA Group. PTBA Group showed significantly higher patent hepatic veins (*P* = .0377) and membranous obstruction rather than thrombotic/segmental obstruction (*P* = .0024) compared with TIPSS Group. TIPSS Group showed significantly lower centrally stenosis/obstruction (approaching hepatic vein confluence/IVC) and higher peripheral stenosis/obstruction (approaching hepatic venules/sinusoids) compared with PTBA Group (*P* < .0001). The locations of hepatic veins involvement and the percent of accessory hepatic veins involvement showed no statistically significant difference between 2 groups (*P* > .05).

**Table 2:**
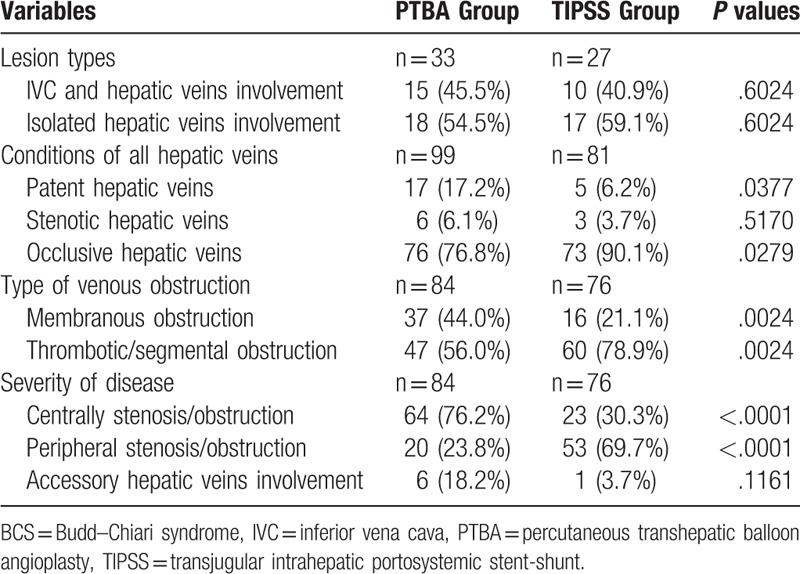
Classification of BCS.

### 3.3 Interventional procedures parameters of PTBA

There were 31 cases of success and 2 cases failed in procedure in PTBA Group, with a success rate of 93.9%. Three hepatic veins occlusion with a large number of collateral circulations were shown in 1 patient via percutaneous transhepatic angiography, the guide wire failed to pass through the hepatic veins. The other patient showed excessive thrombus in the occlusive right hepatic vein, catheter fail to pass through the right hepatic vein. Thirty-seven balloon catheters were used for primary dilation of hepatic veins, and 14 balloon catheters were used for second dilation due to restenosis or reocclusion of hepatic veins during follow up. The mean diameter or mean length of balloon showed no significant difference between primary and second dilation (Table 3). One stent was implanted in right hepatic vein after PTBA, and 1 patient underwent catheter direct thrombolysis due to thrombosis in right hepatic vein during follow up. Of the 15 patients with IVC involvement, 6 patients underwent balloon dilation (diameter range 15 to 30 mm) for IVC, including 2 patients treated by retrivable stent filters. All these retrivable stents filters were withdrawn after successful thrombolysis.

**Table 3:**
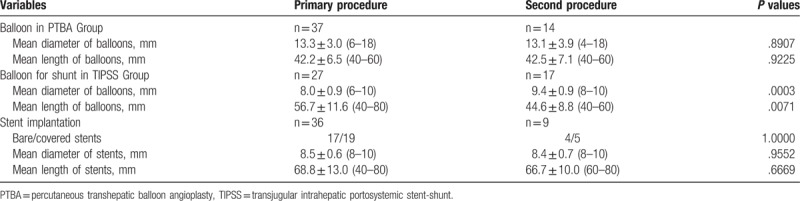
Measurement of balloons and stents.

### 3.4 Interventional procedures parameters of TIPSS

There was no failure or death in TIPSS Group, the success rate was as high as 100%. The catheter failed to enter the stent after repeated attempts in only 1 patient with intrahepatic shunt re-occlusion. However, successful balloon dilation (10 mm∗4 cm) of the intrahepatic shunt performed during second attempt later. Two patients received additional stenting in hepatic veins, and 1 patient received stenting in occlusive IVC. Each patient underwent balloon dilation during TIPSS, and 17 patients underwent second balloon dilation for stenotic or occlusive shunt stents. Interestingly, balloon catheters with significant increased diameter and decreased length were used for second dilation if compared with primary dilation. Thirty-six stents (17 bare and 19 covered stents) was implanted during TIPSS, and 9 stents (4 bare and 5 covered stents) were used for stenotic or occlusive shunt stents after balloon dilation (Table 3). The mean portal vein pressure before procedure was 37.0 ± 6.2 mm H_2_O, which decreased significantly after stenting (18.5 ± 2.2, *P* = .0399). One patient with IVC involvement underwent balloon dilation (20 mm in diameter) during TIPSS procedure. During the follow-up, 6 patients with color Doppler ultrasound suspected the stenosis or occlusion of the shunts and confirmed its patency 9 times angiographies of shunts. Besides, 6 patients underwent gastric coronary veins embolization for hemorrhage of digestive tract, and 2 patients were treated with catheter direct thrombolysis for shunt thrombosis.

### 3.5 Perioperative complications

Perioperative death was found in 1 patient with mixed type of BCS combined with cavernous transformation of the portal vein. Repeated hemorrhage still showed after successful PTBA with 16 mm∗4 cm balloon for right and middle hepatic veins, and patient died of hemorrhagic shock after transfer to intensive care unit. One patient developed skin urticaria after PTBA and was given dexamethasone 10 mg intravenously to relieve symptoms. One patient had a small amount of hematuria due to oral warfarin, and the symptoms disappeared after drug withdrawal. No death was found in TIPSS Group. Only 1 patient showed acute thrombosis in shunt stent 3 days later, thrombus completely resolved after catheter direct thrombolysis. Hematochezia was found in 1 patient 5 days after TIPSS due to oral take of warfarin, symptoms gradually improved after drug withdrawal. Other severe complications such as massive bleeding, rupture of vein, pulmonary embolism, had not been observed in both groups.

### 3.6 Follow up

**Figure 4:**
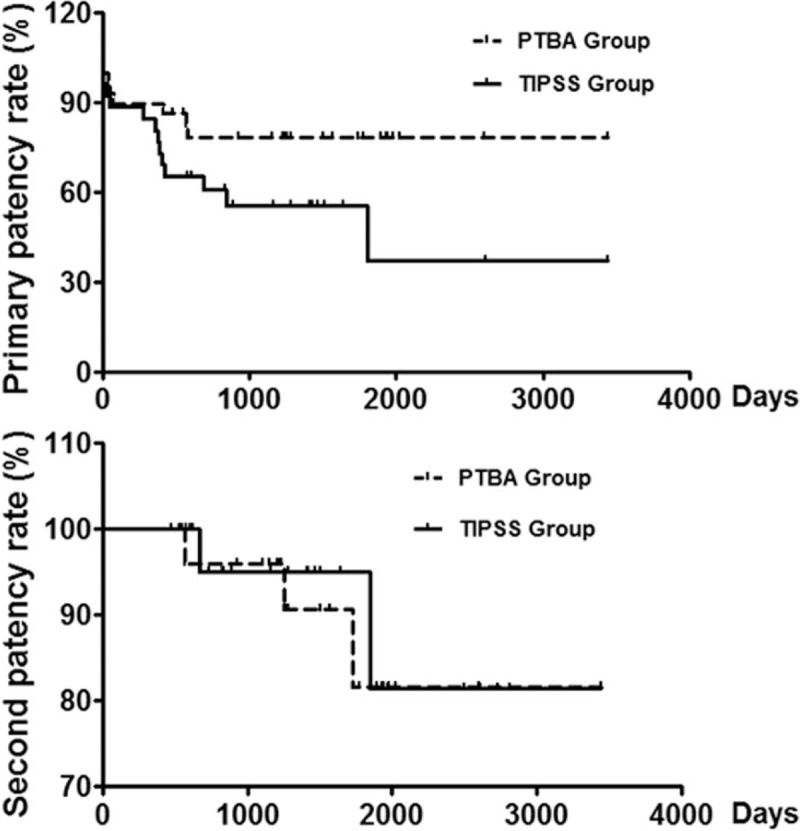
Patency rate of hepatic vein and shunt. PTBA Group showed a higher primary patency rate of hepatic vein than that of shunt in TIPSS Group. There was no significant difference of second patency between 2 groups. PTBA = percutaneous transhepatic balloon angioplasty, TIPSS = transjugular intrahepatic portosystemic stent-shunt.

One death and 2 patients with failure balloon dilation were excluded from follow up in PTBA Group. There was 1 and 3 patient's loss, with follow up rate of 87.9% and 88.9% in PTBA Group and TIPSS Group, respectively. Twenty-six patients (89.7%) and 21 patients (87.5%) were clinically cured in PTBA Group and TIPSS Group, respectively (*P* = 1.0000). As shown in Table 4 and Figure 4, the primary patency rates were 89.7%, 79.3% and 79.3% for short-term, mid-term and long-term in PTBA Group, which were significantly higher than TIPSS Group for long-term follow up (*P* = .0408). The second patency rates were 100.0%, 96.6%, and 96.6% for short-term, mid-term and long-term in PTBA Group, which were similar to TIPSS Group (*P* = 1.0000).

**Table 4:**
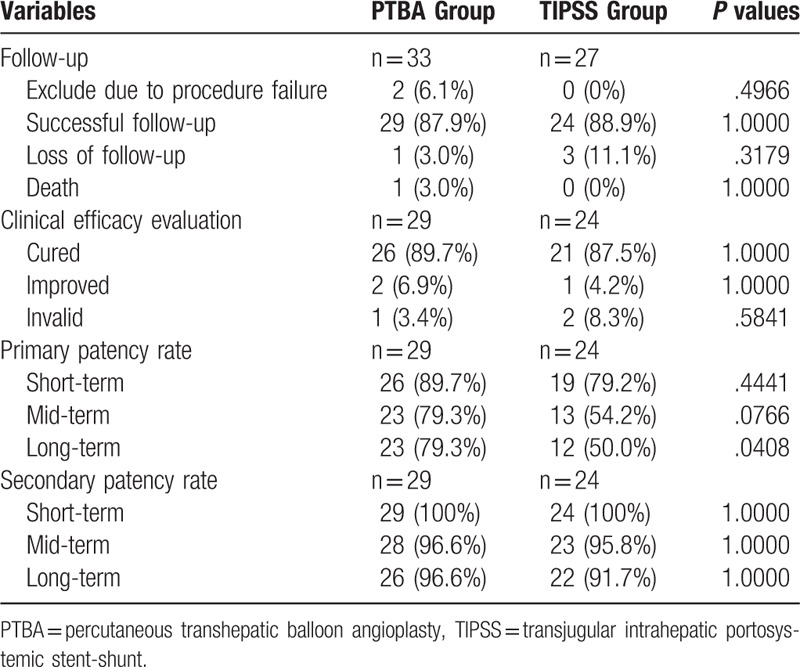
Follow-up and curative effect analysis.

## 4 Discussions

The interventional treatments of hepatic vein lesions in BCS mainly include PTBA, endovascular stent implantation, and TIPSS. Since initial success in balloon angioplasty in 1974^[16]^ and stent placement in 1990,^[17]^ interventional treatments have proven efficacy for BCS. Although interventional treatments have become the preferred treatment of BCS, restenosis seriously affects its clinical outcomes. In hepatic vein type and mixed type BCS, hepatic vein stenosis or occlusive disease leads to hepatic stasis and cirrhosis, presenting as portal hypertension syndrome. It is the key and difficult point of interventional therapy. Compared with western countries, most of BCS in China is mixed type of hepatic vein and IVC involved simultaneously.^[3]^ PTBA and stenting are the main treatments with better prognosis in China. Our results showed that hepatic venous PTBA and TIPSS are safe and effective for BCS with hepatic venous lesions. Hepatic vein and TIPSS shunt patency rate is high; the second patency rate was as high as 96.6% and 91.7%, respectively.

In order to shorten the operation time and avoid excessive X ray radiation, there is no need to canalize all occluded hepatic veins, if successful recanalization of 1 (accessory) hepatic vein can satisfy the normal blood flow of the liver. Percutaneous transhepatic approach should be used immediately when femoral or jugular vein cannulation is difficult, although more complications may relate.^[10]^ TIPSS should be performed to reduce the pressure of portal vein and restore normal liver function if 3 hepatic veins and accessory hepatic veins are obstructed extensively. TIPSS can be used for the treatment of patients with 3 veins and accessory hepatic veins involvement, and patients with recurrent and failure PTBA. This study included 1 failure PTBA and 1 recurrent patient after PTBA for hepatic veins, which treated in other centers.

The balloon diameter selection for hepatic vein is still lack of unified standards. Early clinical experience shows that high recurrence rate was found after the use of 12 mm balloon catheter. Nowadays, a larger diameter balloon expansion, such as 14 to 16 mm or even 18 to 20 mm balloon, was performed. Few stents were used in our study, which was significantly lower than reported in the literature.^[12]^ This may be related to the following factors: the fully expansion of hepatic veins by larger diameter balloon to effectively reduce the vessel wall retraction; use of TIPSS rather than PTBA recanalization for long segmental occlusion by careful selection of the target hepatic vein, thereby reducing the use of stents. Recanalization long segmental occlusion is always difficult with a high failure and reobstructive rate.^[10,18]^ Currently, TIPSS has been used as the preferred treatment for BCS with long-segment obstruction of the hepatic vein in most institutions,^[15,19]^ and accessory hepatic vein recanalization also reported.^[20]^

In addition, complex treatments were used in this study to reduce the incidence of restenosis or occlusion, included catheter direct thrombolysis, stent placement, and retrieval stent placement for IVC involvement. Catheter-direct thrombolysis was used for BCS patients with fresh thrombus in hepatic veins or IVC,^[21,22]^ and retrieval stent placement was used in 2 cases to prevent pulmonary embolism and compress bolt in IVC.^[23–25]^

There are several limitations in this study. This is a retrospective study performed in single center, and a multicenter prospective study can be more clinically instructive. Sonography of TIPSS is poorly sensitive for diagnosing stenosis leading to significant portal hypertension recurrence. In conclusion, PTBA and TIPSS are safe and effective in the treatment of BCS with hepatic veins involvement, with an excellent long-term patency rate of hepatic vein and TIPSS shunt. TIPSS can be used to treat patients with all 3 hepatic veins lesion and failure PTBA.

## Author contributions

**Conceptualization:** Jianzhuang Ren.

**Data curation:** Yonghua Bi, Hongmei Chen, Pengxu Ding, Pengli Zhou, Xinwei Han, Jianzhuang Ren.

**Formal analysis:** Yonghua Bi, Xinwei Han.

**Funding acquisition:** Yonghua Bi.

**Investigation:** Yonghua Bi, Hongmei Chen, Pengxu Ding, Pengli Zhou.

**Methodology:** Yonghua Bi, Hongmei Chen, Pengli Zhou, Jianzhuang Ren.

**Project administration:** Xinwei Han, Jianzhuang Ren.

**Resources:** Yonghua Bi, Hongmei Chen.

**Software:** Yonghua Bi, Pengxu Ding.

**Supervision:** Pengli Zhou, Xinwei Han, Jianzhuang Ren.

**Visualization:** Xinwei Han.

**Writing – original draft:** Yonghua Bi, Hongmei Chen.

**Writing – review & editing:** Pengxu Ding, Pengli Zhou, Xinwei Han, Jianzhuang Ren.
